# Crystal structure and Hirshfeld surface analysis of (*E*)-3-(3-iodo­phen­yl)-1-(4-iodo­phen­yl)prop-2-en-1-one

**DOI:** 10.1107/S2056989019016402

**Published:** 2020-01-01

**Authors:** Kieran J. Spruce, Charlie L. Hall, Jason Potticary, Natalie E. Pridmore, Matthew E. Cremeens, Gemma D. D’ambruoso, Masaomi Matsumoto, Gabrielle I. Warren, Stephen D. Warren, Simon R. Hall

**Affiliations:** a School of Chemistry, University of Bristol, Cantock’s Close, Bristol, England, BS8 1TS, England; b Department of Chemistry & Biochemistry, Gonzaga University, 502 E Boone Ave, Spokane, WA 99258, USA

**Keywords:** crystal structure, *E* configuration, iodo­phenyl ring, chalcone, (*E*)-3-(3-iodo­phen­yl)-1-(4-iodo­phen­yl)prop-2-en-1-one

## Abstract

The title compound, C_15_H_10_I_2_O, is a halogenated chalcone formed from two iodine substituted rings, one *para*-substituted and the other *meta*-substituted, linked through a prop-2-en-1-one spacer. In the mol­ecule, the mean planes of the 3-iodo­phenyl and the 4-iodo­phenyl groups are twisted by 46.51 (15)°. The calculated electrostatic potential surfaces show the presence of σ-holes on both substituted iodines. In the crystal, the mol­ecules are linked through type II halogen bonds, forming a sheet structure parallel to the *bc* plane. Between the sheets, weak inter­molecular C—H⋯π inter­actions are observed. Hirshfeld surface analysis showed that the most significant contacts in the structure are C⋯H/H⋯C (31.9%), followed by H⋯H (21.4%), I⋯H/H⋯I (18.4%). I⋯I (14.5%) and O⋯H/H⋯O (8.1%).

## Abstract 2

The title compound, C_15_H_10_I_2_O, is a halogenated chalcone formed from two iodine substituted rings, one *para*-substituted and the other *meta*-substituted, linked through a prop-2-en-1-one spacer. The crystal structure shows the mol­ecules contain halogen bonds which are always between equivalent iodine atoms, either *para*–*para* or *meta*–*meta*.

## Chemical context   

Chalcones are aromatic ketones which have shown potential as anti­bacterial, anti­fungal and anti-inflammatory agents (D’silva *et al.*, 2011 ▸). These mol­ecules are essential to the biosynthesis of flavonoids through a conjugate ring-closure to form flavone and have also attracted attention for their potential use in opto- and organic electronics (Shetty *et al.*, 2016 ▸, 2017 ▸). As a family of mol­ecules, substituted chalcones can be readily synthesized *via* a Claisen–Schmidt condensation reaction between an appropriately functionalized aceto­phenone and benzaldehyde. Substitutions on each of the benzene rings are currently being investigated in order to inter­rogate how the electronic properties of the crystal are altered. The iodo-substituted rings present in the title compound allows for the formation of iodine channels in the crystal, a conformation which may afford a change in the crystal’s electrical properties.
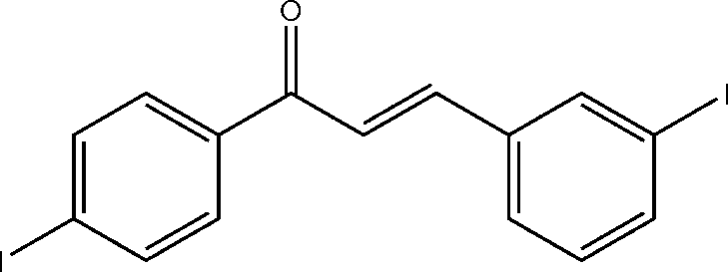


## Structural commentary   

**Figure 1:**
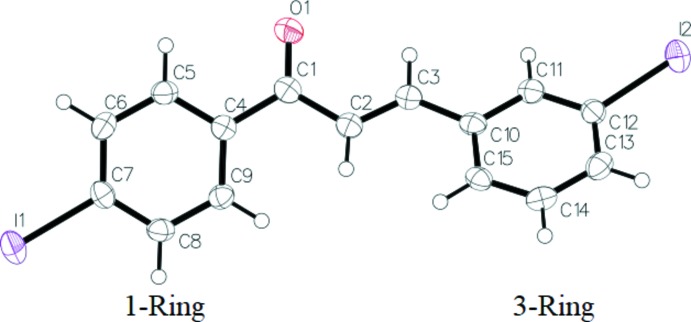
The mol­ecular structure of the title compound, showing the atom labelling and displacement ellipsoids drawn at the 50% probability level.

The title compound comprises two aromatic rings, 4-iodo­phenyl (1-Ring) and 3-iodo­phenyl (3-Ring), which are connected, respectively, to atoms C1 and C3 of the –CO—CH=CH– enone bridge (Fig. 1 ▸). The backbone torsion angles are C5—C4—C1—C2 = 151.6 (4)°, C4—C1—C2—C3 = 171.9 (4)°, C1—C2—C3—C10 = 176.4 (4)° and C2—C3—C10—C11 = 170.4 (5)°. The mean planes of the 3-iodo­phenyl and 4-iodo­phenyl groups are twisted by 46.51 (15)° relative to each other. The H atoms of the propenone group are *trans*-configured.

## Supra­molecular features   

**Figure 2:**
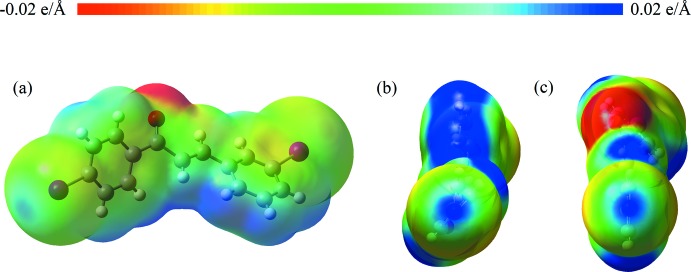
Electrostatic potential mapped onto an electron density isosurface with isovalue 0.02 e Å^−3^, calculated using B3LYP at the LANL2DZ level. Red and blue regions show negative and positive electric potentials, respectively. (*a*) shows the potential of the substituted chalcone mol­ecule. (*b*) and (*c*) show the σ-holes on 1-Ring and 3-Ring, respectively.

**Figure 3:**
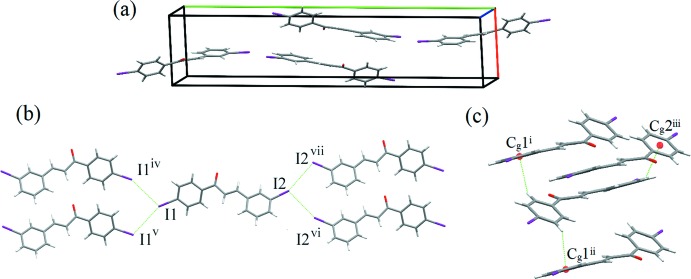
(*a*) A packing diagram of the title compound in the unit cell. Red, green and blue axes indicate *a*, *b* and *c*, respectively. (*b*) *Meta*–*meta* and *para*–*para* halogen bonds indicated by dashed lines. (*c*) Three weak C—H⋯π inter­actions (dashed lines; C5—H5⋯*Cg*1^i^, C8—H8⋯*Cg*1^ii^ and C14—H14⋯*Cg*2^iii^). *Cg*1 and *Cg*2 are the centroids of the C10–C15 and C4–C9 rings, respectively. [Symmetry codes: (i) 1 − *x*, 1 − *y*, 1 − *z*; (ii) 2 − *x*, 1 − *y*, 2 − *z*; (iii) 1 − *x*, 1 − *y*, 2 − *z*; (iv) *x*, 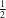 − *y*, −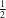 + *z*; (v) *x*, 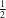 − *y*, 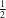 + *z*; (vi) *x*, 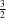 − *y*, 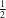 + *z*; (vii) *x*, 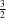 − *y*, −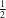 + *z.*.]

Electrostatic potential surfaces [Fig. 2 ▸(*a*)–(*c*)] show the presence of σ-holes on both substituted iodines, I1 and I2, which allow for halogen bonding of a bifurcated type II. Partial packing diagrams are shown in Fig. 3 ▸(*a*)–(*c*). Inter­estingly, these halogen bonds form exclusively between equivalent iodine atoms, either *para*–*para* or *meta*–*meta*. The geometries of the halogen bonds are I1⋯I1^iv^ = 4.0980 (9) Å, C7—I1⋯I1^iv^ = 113.85 (13)°, I1⋯I1^v^ =4.0980 (9) Å and C7—I⋯I1^v^ = 154.47 (13)° for the *para*–*para* I⋯I bonds, and I2⋯I2^vi^ = 3.9805 (8) Å, C12—I2⋯I2^vi^ = 108.20 (13)°, I2⋯I2^vii^ = 3.9805 (8) Å and C12—I2⋯I2^vii^ = 157.30 (13)° for the *meta*–*meta* I⋯I bonds [symmetry codes: (iv) *x*, 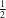 − *y*, −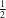 + *z*; (v) *x*, 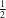 − *y*, 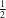 + *z*; (vi) *x*, 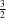 − *y*, 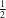 + *z*; (vii) *x*, 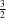 − *y*, −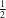 + *z*]. A sheet structure is formed parallel to the *bc* plane. There are also three weak C—H⋯π inter­actions (Table 1 ▸) between the sheets.

**Figure 4:**
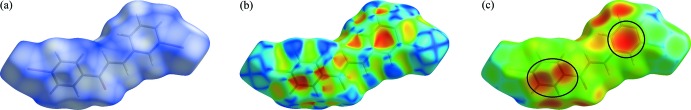
Hirshfeld surfaces of the title compound, mapped with (*a*) *d*_norm_, where white regions represent inter­actions equal to, and blue regions represent inter­actions shorter than the sum of their van der Waals radii, (*b*) the shape-index, and (*c*) *d*_e_, where the circled areas indicate the C—H⋯π inter­actions.

**Figure 5:**
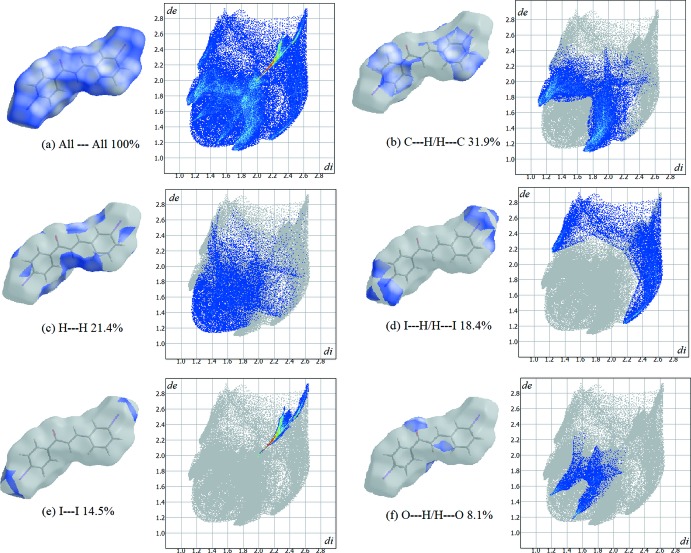
Hirshfeld surfaces and fingerprint plots showing percentage of contacts of (*a*) all inter­actions, (*b*) C⋯H/H⋯C, (*c*) H⋯H, (*d*) I⋯H/H⋯I, (*e*) I⋯I and (*f*) O⋯H/H⋯O. inter­actions.

Hirshfeld surfaces, mapped over *d*_norm_, shape-index and *d*_e_, and two-dimensional fingerprint plots of the title compound were calculated using *CrystalExplorer17.5* (Turner *et al.*, 2017 ▸). Hirshfeld surfaces [Fig. 4 ▸(*a*) and (*c*)] highlight the relationship between the contact distance and the van der Waals radii (Venkatesan *et al.*, 2016 ▸). The Hirshfeld surface mapped over the shape-index [Fig. 4 ▸(*b*)] shows depressions on both 1-Ring and 3-Ring, which is indicative of C—H⋯π inter­actions. Two-dimensional fingerprint plots are used to illustrate the inter­molecular contacts between mol­ecules within the crystal structure. The fingerprint plots of all significant inter­actions are shown in Fig. 5 ▸(*a*)–(*f*). C⋯H/H⋯C contacts [Fig. 5 ▸(*b*)] make the largest contribution (31.9%) and show a pair of spikes at *d*_e_ + *d*_i_ = ∼2.8 Å, representative of inter­molecular C—H⋯π inter­actions. The O⋯H/H⋯O plot also contains a pair of spikes at *d*_e_ + *d*_i_ = ∼2.7 Å [Fig. 5 ▸(*f*)]. The negligible contributions from other contacts, not included in Fig. 5 ▸, are as follows: C⋯C (3.1%), C⋯O/O⋯C (2.1%) and C⋯I/I⋯C (0.5%).

## Database survey   

A survey of the Cambridge Structural Database (CSD; Groom *et al.*, 2016 ▸) showed that existing similar structures include (2*E*)-1-(4-bromo­phen­yl)-3-(4-fluoro­phen­yl)prop-2-en-1-one (refcode NURCIN; Dutkiewicz *et al.*, 2010 ▸), 1-(4-bromo­phen­yl)-3-(4-chloro­phen­yl)prop-2-en-1-one (LEPYIP; Yang *et al.*, 2006 ▸), 1,3-bis­(4-bromo­phen­yl)prop-2-en-1-one (LEHROG; Ng *et al.*, 2006 ▸), (*E*)-1-(4-bromo­phen­yl)-3-(4-iodo­phen­yl)prop-2-en-1-one (IWALAV; Zainuri *et al.*, 2017 ▸) and 3-(3-bromo­phen­yl)-1-(4-bromo­phen­yl)prop-2-en-1-one (ODEDEH; Teh *et al.*, 2006 ▸). Four compounds (NURCIN, LEPYIP, LEHROG and IWALAV) contain only *para*-substituted rings. Within these structures, halogen bonds exist only between bromine and iodine species, and never between equivalent halogens. 3-(3-Bromo­phen­yl)-1-(4-bromo­phen­yl)prop-2-en-1-one contains one *meta*-substituted ring and one *para*-substituted ring: each halogen bond exists between rings with the same substitution, either *para*–*para* or *meta*–*meta*, as seen in the title compound.

## Synthesis and crystallization   

4′-Iodo­aceto­phenone (0.773 g, 3.14 mmol), 3-iodo­benzaldehyde (0.697 g, 3.00), anhydrous zinc chloride (0.615 g, 4.51 mmol) and absolute ethanol (1.5 ml) were added to a microwave vessel with a stir bar. Using a microwave reactor, the reaction mixture was heated to 468 K for 15 minutes. Upon cooling the reaction, yellowish solids were collected by vacuum filtration and washed with 95% ethanol. The resulting solid was recrystallized from 95% ethanol (0.603 g, 44% yield, yellow crystals, m.p. 442.5–443.7 K). ^1^H NMR (400 MHz, DMSO-*d*_6_, referenced to TMS): *δ* (ppm) 8.37 (1H, *s*), 8.0–7.94 (5H, *m*), 7.88 (1H, *d*, *J* = 8 Hz), 7.81 (1H, *d*, *J* = 8 Hz), 7.68 (1H, *d*, *J* = 16 Hz), 7.26 (1H, *t*, *J* = 8 Hz). ^13^C NMR (100 MHz, DMSO-*d*_6_, referenced to solvent, 39.52 ppm): *δ* (ppm) 188.44, 142.75, 139.04, 137.72, 136.95, 136.66, 136.61, 130.89, 130.38, 128.73, 122.81, 102.14, 95.57. Single crystals suitable for X-ray diffraction were obtained by the slow evaporation technique from an acetone solution at room temperature.

## Refinement   

Crystal data, data collection and structure refinement details are summarized in Table 2 ▸. H atoms were positioned geometrically (C—H = 0.95 Å) and refined using a riding model with *U*_iso_(H) = 1.2*U*_eq_(C).

## Supplementary Material

Crystal structure: contains datablock(s) I. DOI: 10.1107/S2056989019016402/is5526sup1.cif

Structure factors: contains datablock(s) I. DOI: 10.1107/S2056989019016402/is5526Isup2.hkl

Supporting information file. DOI: 10.1107/S2056989019016402/is5526Isup5.mol

13C NMR of the synthesised product. DOI: 10.1107/S2056989019016402/is5526sup3.pdf

1H NMR of the synthesised product. DOI: 10.1107/S2056989019016402/is5526sup4.pdf

Supporting information file. DOI: 10.1107/S2056989019016402/is5526Isup6.cml

CCDC reference: 1970266

Additional supporting information:  crystallographic information; 3D view; checkCIF report

## Figures and Tables

**Table 1:** Hydrogen-bond geometry (Å, °) *Cg*1 and *Cg*2 are the centroids of the C10–C15 and C4–C9 rings, respectively.

*D*—H⋯*A*	*D*—H	H⋯*A*	*D*⋯*A*	*D*—H⋯*A*
C5—H5⋯*Cg*1^i^	0.95	2.78	3.406 (5)	124
C8—H8⋯*Cg*1^ii^	0.95	2.85	3.491 (5)	126
C14—H14⋯*Cg*2^iii^	0.95	2.77	3.440 (5)	129

Symmetry codes: (i) 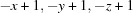; (ii) 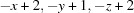; (iii) 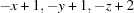.

**Table 2:** Experimental details

Crystal data	
Chemical formula	C_15_H_10_I_2_O
*M*_r_	460.03
Crystal system, space group	Monoclinic, *P*2_1_/*c*
Temperature (K)	200
*a*, *b*, *c* (Å)	7.2650 (7), 32.864 (3), 5.8446 (6)
β (°)	92.277 (2)
*V* (Å^3^)	1394.3 (2)
*Z*	4
Radiation type	Mo *K*α
μ (mm^−1^)	4.50
Crystal size (mm)	0.57 × 0.29 × 0.08

Data collection	
Diffractometer	Bruker APEXII kappa CCD area detector
Absorption correction	Numerical (*SADABS*; Bruker, 2016 ▸)
*T*_min_, *T*_max_	0.065, 0.189
No. of measured, independent and observed [*I* > 2σ(*I*)] reflections	18346, 3215, 2960
*R*_int_	0.021
(sin θ/λ)_max_ (Å^−1^)	0.650

Refinement	
*R*[*F* ^2^ > 2σ(*F* ^2^)], *wR*(*F* ^2^), *S*	0.037, 0.073, 1.27
No. of reflections	3215
No. of parameters	164
H-atom treatment	H-atom parameters constrained
Δρ_max_, Δρ_min_ (e Å^−3^)	1.14, −1.31

Computer programs: *SAINT* (Bruker, 2016 ▸), *SHELXT* (Sheldrick, 2015*a* ▸), *SHELXL2018* (Sheldrick, 2015*b* ▸) and *OLEX2* (Dolomanov *et al.*, 2009 ▸).

## supplementary crystallographic information

### Crystal data

**Table:**

C_15_H_10_I_2_O	*F*(000) = 856
*M**_r_* = 460.03	*D*_x_ = 2.191 Mg m^−^^3^
Monoclinic, *P*2_1_/*c*	Mo *K*α radiation, λ = 0.71073 Å
*a* = 7.2650 (7) Å	Cell parameters from 8513 reflections
*b* = 32.864 (3) Å	θ = 2.5–27.5°
*c* = 5.8446 (6) Å	µ = 4.50 mm^−^^1^
β = 92.277 (2)°	*T* = 200 K
*V* = 1394.3 (2) Å^3^	Plate, clear colourless
*Z* = 4	0.57 × 0.29 × 0.08 mm

### Data collection

**Table:**

Bruker APEXII kappa CCD area detector diffractometer	3215 independent reflections
Radiation source: fine-focus sealed tube	2960 reflections with *I* > 2σ(*I*)
Graphite monochromator	*R*_int_ = 0.021
φ and ω scans	θ_max_ = 27.5°, θ_min_ = 2.5°
Absorption correction: numerical (SADABS; Bruker, 2016)	*h* = −9→9
*T*_min_ = 0.065, *T*_max_ = 0.189	*k* = −42→37
18346 measured reflections	*l* = −7→7

### Refinement

**Table:**

Refinement on *F*^2^	Hydrogen site location: inferred from neighbouring sites
Least-squares matrix: full	H-atom parameters constrained
*R*[*F*^2^ > 2σ(*F*^2^)] = 0.037	*w* = 1/[σ^2^(*F*_o_^2^) + (0.0034*P*)^2^ + 6.4729*P*] where *P* = (*F*_o_^2^ + 2*F*_c_^2^)/3
*wR*(*F*^2^) = 0.073	(Δ/σ)_max_ = 0.001
*S* = 1.27	Δρ_max_ = 1.14 e Å^−^^3^
3215 reflections	Δρ_min_ = −1.30 e Å^−^^3^
164 parameters	Extinction correction: SHELXL2018 (Sheldrick, 2015*b*), Fc^*^=kFc[1+0.001xFc^2^λ^3^/sin(2θ)]^-1/4^
0 restraints	Extinction coefficient: 0.00062 (7)
Primary atom site location: iterative	

### Special details

**Table:**

Geometry. All esds (except the esd in the dihedral angle between two l.s. planes) are estimated using the full covariance matrix. The cell esds are taken into account individually in the estimation of esds in distances, angles and torsion angles; correlations between esds in cell parameters are only used when they are defined by crystal symmetry. An approximate (isotropic) treatment of cell esds is used for estimating esds involving l.s. planes.

### Fractional atomic coordinates and isotropic or equivalent isotropic displacement parameters (Å^2^)

**Table:**

	*x*	*y*	*z*	*U*_iso_*/*U*_eq_	
C4	0.8336 (6)	0.43230 (13)	0.5707 (7)	0.0250 (9)	
C9	0.9114 (6)	0.42521 (14)	0.7892 (8)	0.0278 (9)	
H9	0.939830	0.447424	0.888449	0.033*	
C8	0.9474 (6)	0.38548 (14)	0.8617 (8)	0.0289 (9)	
H8	1.003866	0.380553	1.008533	0.035*	
C7	0.9004 (6)	0.35322 (14)	0.7183 (8)	0.0292 (9)	
C6	0.8265 (6)	0.35974 (14)	0.4992 (8)	0.0298 (10)	
H6	0.797488	0.337429	0.400879	0.036*	
C5	0.7955 (6)	0.39954 (14)	0.4252 (8)	0.0285 (9)	
H5	0.747935	0.404397	0.273958	0.034*	
C1	0.7892 (6)	0.47421 (14)	0.4874 (8)	0.0284 (9)	
C2	0.7447 (6)	0.50560 (14)	0.6605 (8)	0.0294 (9)	
H2	0.729579	0.497837	0.815221	0.035*	
C3	0.7258 (6)	0.54438 (13)	0.5999 (8)	0.0268 (9)	
H3	0.749373	0.550781	0.445302	0.032*	
C10	0.6729 (6)	0.57825 (13)	0.7450 (7)	0.0251 (9)	
C11	0.6878 (6)	0.61791 (13)	0.6589 (8)	0.0273 (9)	
H11	0.740192	0.622248	0.514565	0.033*	
C12	0.6264 (6)	0.65086 (13)	0.7836 (8)	0.0289 (9)	
C13	0.5540 (6)	0.64548 (15)	0.9989 (8)	0.0322 (10)	
H13	0.513741	0.668101	1.084900	0.039*	
C14	0.5424 (6)	0.60607 (15)	1.0845 (8)	0.0311 (10)	
H14	0.493446	0.601952	1.230991	0.037*	
C15	0.6003 (6)	0.57271 (14)	0.9618 (8)	0.0280 (9)	
H15	0.590951	0.546131	1.024224	0.034*	
I1	0.92866 (6)	0.29371 (2)	0.84509 (7)	0.04484 (12)	
I2	0.63173 (6)	0.70888 (2)	0.63477 (6)	0.04434 (12)	
O1	0.7830 (5)	0.48192 (10)	0.2821 (6)	0.0376 (8)	

### Atomic displacement parameters (Å^2^)

**Table:**

	*U*^11^	*U*^22^	*U*^33^	*U*^12^	*U*^13^	*U*^23^
C4	0.022 (2)	0.027 (2)	0.026 (2)	0.0011 (17)	0.0023 (16)	0.0009 (17)
C9	0.027 (2)	0.028 (2)	0.028 (2)	−0.0020 (18)	−0.0031 (18)	−0.0029 (17)
C8	0.028 (2)	0.034 (2)	0.024 (2)	0.0032 (19)	−0.0031 (17)	0.0017 (18)
C7	0.027 (2)	0.026 (2)	0.034 (2)	0.0048 (18)	0.0022 (18)	0.0026 (18)
C6	0.029 (2)	0.029 (2)	0.031 (2)	0.0003 (18)	−0.0001 (18)	−0.0077 (18)
C5	0.028 (2)	0.033 (2)	0.024 (2)	0.0036 (18)	−0.0010 (17)	−0.0009 (17)
C1	0.023 (2)	0.031 (2)	0.031 (2)	0.0005 (17)	0.0008 (18)	0.0024 (18)
C2	0.033 (2)	0.028 (2)	0.027 (2)	−0.0004 (18)	0.0022 (18)	0.0019 (17)
C3	0.026 (2)	0.029 (2)	0.026 (2)	−0.0005 (18)	0.0003 (17)	0.0016 (17)
C10	0.021 (2)	0.030 (2)	0.025 (2)	−0.0016 (17)	−0.0045 (16)	0.0015 (17)
C11	0.027 (2)	0.029 (2)	0.026 (2)	−0.0048 (18)	−0.0012 (17)	0.0022 (17)
C12	0.031 (2)	0.024 (2)	0.031 (2)	−0.0037 (18)	−0.0077 (18)	0.0016 (17)
C13	0.028 (2)	0.035 (2)	0.033 (2)	0.0014 (19)	−0.0018 (19)	−0.0073 (19)
C14	0.028 (2)	0.041 (3)	0.024 (2)	−0.002 (2)	−0.0003 (18)	−0.0015 (19)
C15	0.025 (2)	0.030 (2)	0.028 (2)	0.0001 (18)	−0.0048 (17)	0.0043 (18)
I1	0.0555 (2)	0.02652 (17)	0.0520 (2)	0.00766 (15)	−0.00426 (16)	0.00411 (14)
I2	0.0618 (3)	0.02421 (16)	0.0466 (2)	−0.00405 (15)	−0.00256 (16)	0.00229 (14)
O1	0.051 (2)	0.0340 (18)	0.0278 (17)	0.0045 (16)	0.0029 (15)	0.0051 (14)

### Geometric parameters (Å, º)

**Table:**

C4—C9	1.395 (6)	C2—C3	1.328 (6)
C4—C5	1.393 (6)	C3—H3	0.9500
C4—C1	1.492 (6)	C3—C10	1.460 (6)
C9—H9	0.9500	C10—C11	1.403 (6)
C9—C8	1.394 (6)	C10—C15	1.403 (6)
C8—H8	0.9500	C11—H11	0.9500
C8—C7	1.386 (6)	C11—C12	1.388 (6)
C7—C6	1.386 (6)	C12—C13	1.394 (7)
C7—I1	2.099 (4)	C12—I2	2.097 (4)
C6—H6	0.9500	C13—H13	0.9500
C6—C5	1.393 (6)	C13—C14	1.392 (7)
C5—H5	0.9500	C14—H14	0.9500
C1—C2	1.490 (6)	C14—C15	1.385 (7)
C1—O1	1.225 (5)	C15—H15	0.9500
C2—H2	0.9500		
			
C9—C4—C1	121.8 (4)	C3—C2—H2	119.8
C5—C4—C9	119.5 (4)	C2—C3—H3	116.4
C5—C4—C1	118.6 (4)	C2—C3—C10	127.1 (4)
C4—C9—H9	120.0	C10—C3—H3	116.4
C8—C9—C4	119.9 (4)	C11—C10—C3	118.3 (4)
C8—C9—H9	120.0	C11—C10—C15	118.8 (4)
C9—C8—H8	120.2	C15—C10—C3	122.8 (4)
C7—C8—C9	119.6 (4)	C10—C11—H11	119.8
C7—C8—H8	120.2	C12—C11—C10	120.4 (4)
C8—C7—I1	118.8 (3)	C12—C11—H11	119.8
C6—C7—C8	121.2 (4)	C11—C12—C13	121.0 (4)
C6—C7—I1	120.0 (3)	C11—C12—I2	118.7 (3)
C7—C6—H6	120.5	C13—C12—I2	120.2 (3)
C7—C6—C5	118.9 (4)	C12—C13—H13	120.9
C5—C6—H6	120.5	C14—C13—C12	118.2 (4)
C4—C5—C6	120.7 (4)	C14—C13—H13	120.9
C4—C5—H5	119.6	C13—C14—H14	119.1
C6—C5—H5	119.6	C15—C14—C13	121.8 (4)
C2—C1—C4	117.9 (4)	C15—C14—H14	119.1
O1—C1—C4	120.6 (4)	C10—C15—H15	120.1
O1—C1—C2	121.4 (4)	C14—C15—C10	119.8 (4)
C1—C2—H2	119.8	C14—C15—H15	120.1
C3—C2—C1	120.4 (4)		
			
C4—C9—C8—C7	−1.9 (7)	C2—C3—C10—C11	170.4 (5)
C4—C1—C2—C3	171.9 (4)	C2—C3—C10—C15	−12.5 (7)
C9—C4—C5—C6	2.9 (7)	C3—C10—C11—C12	175.2 (4)
C9—C4—C1—C2	−28.3 (6)	C3—C10—C15—C14	−176.0 (4)
C9—C4—C1—O1	154.5 (5)	C10—C11—C12—C13	1.9 (7)
C9—C8—C7—C6	3.3 (7)	C10—C11—C12—I2	−175.0 (3)
C9—C8—C7—I1	−173.2 (3)	C11—C10—C15—C14	1.0 (6)
C8—C7—C6—C5	−1.5 (7)	C11—C12—C13—C14	−0.9 (7)
C7—C6—C5—C4	−1.6 (7)	C12—C13—C14—C15	0.0 (7)
C5—C4—C9—C8	−1.1 (7)	C13—C14—C15—C10	0.0 (7)
C5—C4—C1—C2	151.6 (4)	C15—C10—C11—C12	−1.9 (6)
C5—C4—C1—O1	−25.6 (7)	I1—C7—C6—C5	175.0 (3)
C1—C4—C9—C8	178.8 (4)	I2—C12—C13—C14	175.9 (3)
C1—C4—C5—C6	−177.0 (4)	O1—C1—C2—C3	−10.9 (7)
C1—C2—C3—C10	176.4 (4)		

### Hydrogen-bond geometry (Å, º)

*Cg*1 and *Cg*2 are the centroids of the C10–C15 and C4–C9 rings, respectively.

**Table:**

*D*—H···*A*	*D*—H	H···*A*	*D*···*A*	*D*—H···*A*
C5—H5···*Cg*1^i^	0.95	2.78	3.406 (5)	124
C8—H8···*Cg*1^ii^	0.95	2.85	3.491 (5)	126
C14—H14···*Cg*2^iii^	0.95	2.77	3.440 (5)	129

Symmetry codes: (i) −*x*+1, −*y*+1, −*z*+1; (ii) −*x*+2, −*y*+1, −*z*+2; (iii) −*x*+1, −*y*+1, −*z*+2.
