# Supplementary material for: Crystal structure and Hirshfeld surface analysis of (E)-3-(3-iodo­phen­yl)-1-(4-iodo­phen­yl)prop-2-en-1-one
Source: Acta Crystallogr E Crystallogr Commun. 2020 Jan 1;76(Pt 1):72–6. doi: 10.1107/S2056989019016402 (PMC6944092; doi:10.1107/S2056989019016402)
Supplement: Supplementary file 4 [file e-76-00072-sup3.pdf]

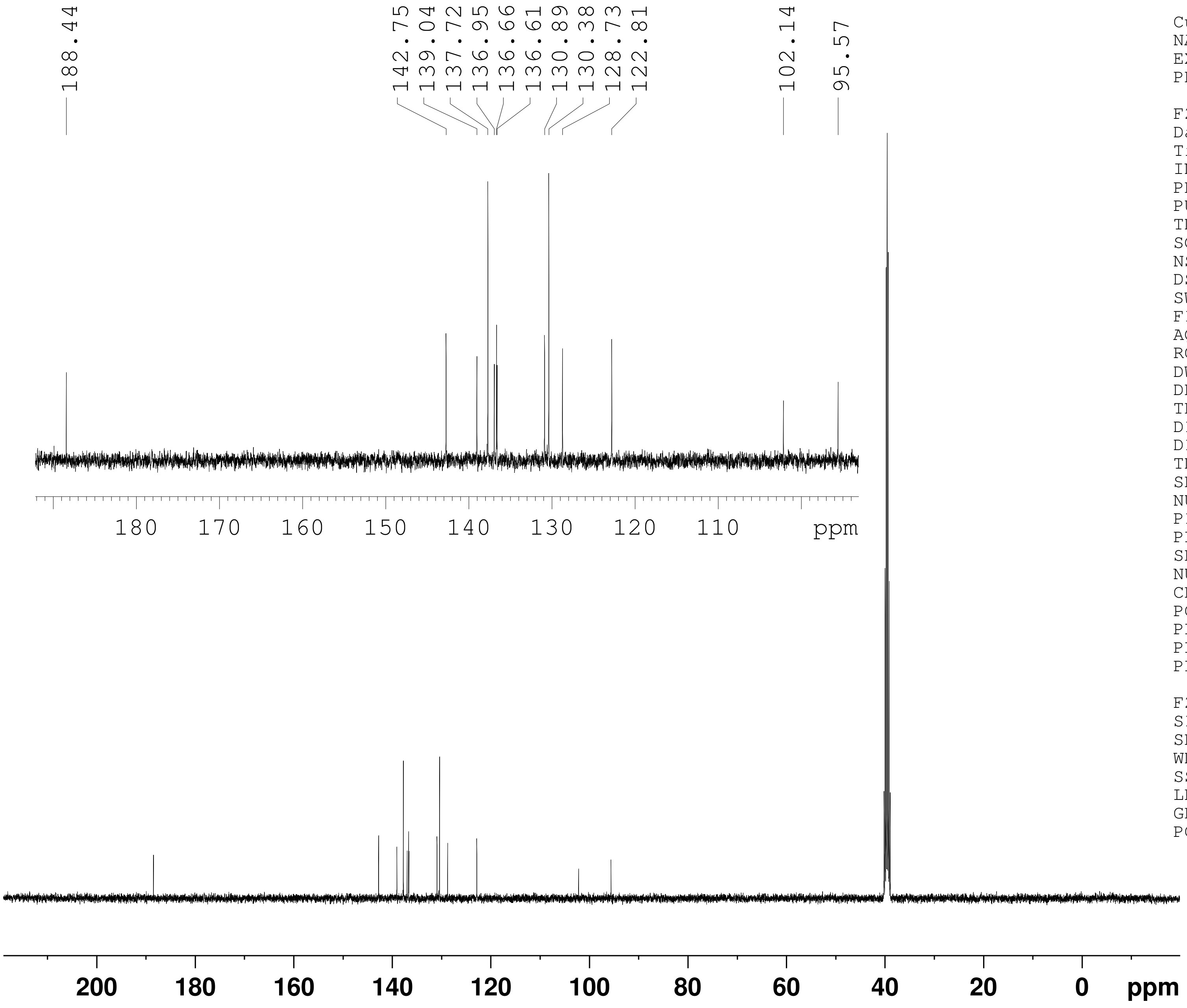

Current Data Parameters

NAME	Ep5m
EXPNO	11
PROCNO	1

F2 - Acquisition Parameters

Date_	20180523
Time	13.35 h
INSTRUM	spect
PROBHD	Z108618_0962 (
PULPROG	zgpg30
TD	65536
SOLVENT	DMSO
NS	128
DS	4
SWH	24038.461 Hz
FIDRES	0.733596 Hz
AQ	1.3631488 sec
RG	202.4
DW	20.800 usec
DE	6.50 usec
TE	295.5 K
D1	2.00000000 sec
D11	0.03000000 sec
TD0	1
SFO1	100.6555216 MHz
NUC1	13C
P1	10.00 usec
PLW1	48.63600159 W
SFO2	400.2616010 MHz
NUC2	1H
CPDPRG[2	waltz16
PCPD2	90.00 usec
PLW2	11.72999954 W
PLW12	0.28382999 W
PLW13	0.14275999 W

F2 - Processing parameters

SI	32768
SF	100.6455052 MHz
WDW	EM
SSB	0
LB	1.00 Hz
GB	0
PC	1.40
