# Supplementary material for: Crystal structure and Hirshfeld surface analysis of (E)-3-(3-iodo­phen­yl)-1-(4-iodo­phen­yl)prop-2-en-1-one
Source: Acta Crystallogr E Crystallogr Commun. 2020 Jan 1;76(Pt 1):72–6. doi: 10.1107/S2056989019016402 (PMC6944092; doi:10.1107/S2056989019016402)
Supplement: Supplementary file 5 [file e-76-00072-sup4.pdf]

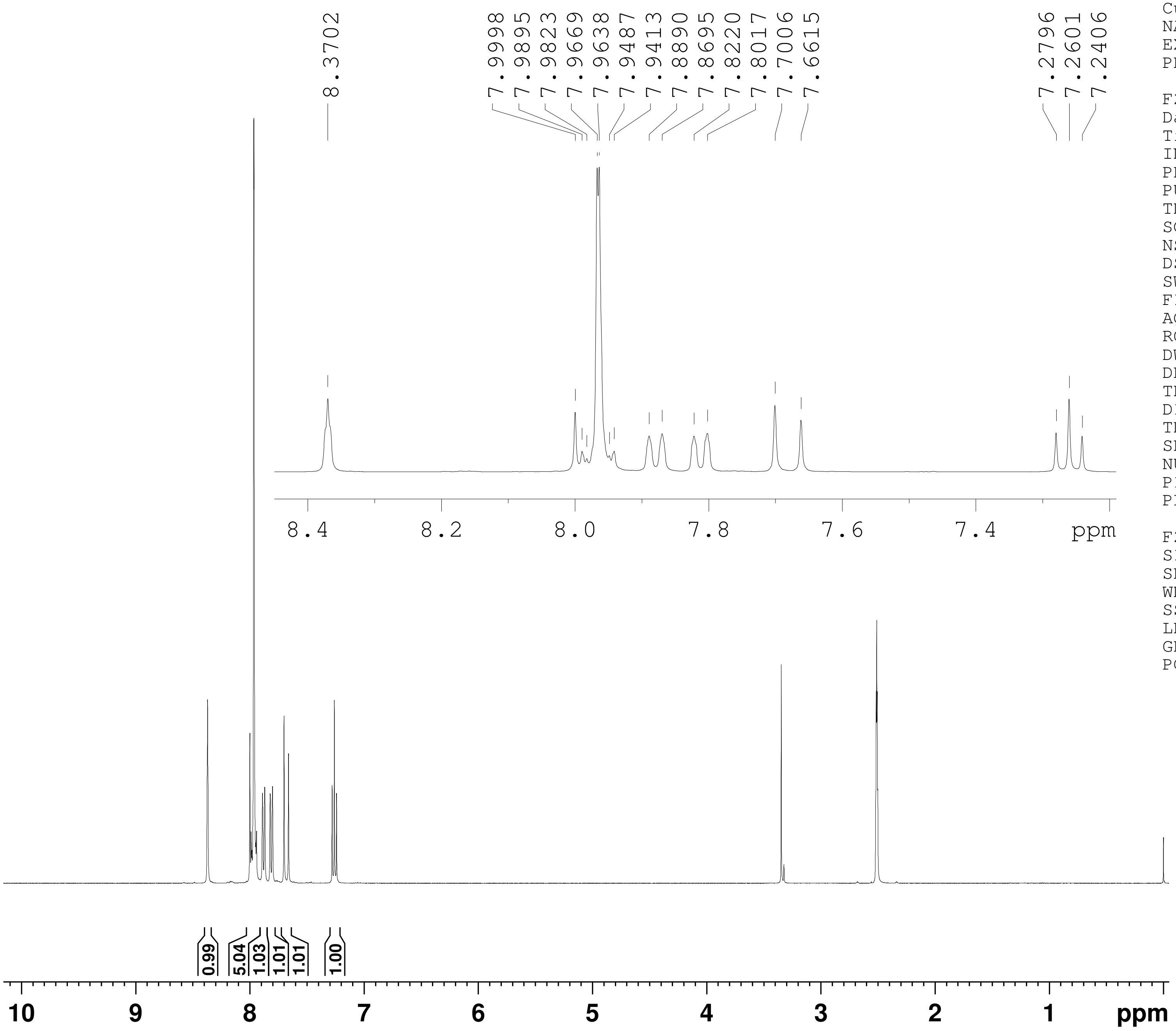

Current Data Parameters	
NAME	Ep5m
EXPNO	10
PROCNO	1
F2 - Acquisition Parameters	
Date_	20180523
Time	13.26 h
INSTRUM	spect
PROBHD	Z108618_0962 (
PULPROG	zg30
TD	65536
SOLVENT	DMSO
NS	16
DS	2
SWH	8012.820 Hz
FIDRES	0.244532 Hz
AQ	4.0894465 sec
RG	143.16
DW	62.400 usec
DE	6.50 usec
TE	294.6 K
D1	1.00000000 sec
TD0	1
SFO1	400.2624716 MHz
NUC1	1H
P1	14.00 usec
PLW1	11.72999954 W
F2 - Processing parameters	
SI	65536
SF	400.2599993 MHz
WDW	EM
SSB	0
LB	0.30 Hz
GB	0
PC	1.00
